# Medical Specialties

**Published:** 1902-06-15

**Authors:** E. I. Backus

**Affiliations:** St. Joseph, Mich.


### MEDICAL SPECIALTIES.

BY E. I. BACKUS, D.D.S., ST. JOSEPH, MICH.

Read before the Southwestern Michigan Dental Society, April 9, 1902.

The rapid development which the several branches of medicine and surgery have undergone in recent years, and the literature that each specialty has produced, almost entitles each one of them to the dignity of a separate science. Divided as the profession of medicine is among so many different specialties, it would be impossible to separate them entirely without impairing their usefulness, for to be successful in any specialty of the science of medicine and surgery requires a more or less comprehensive knowledge of the whole. Since an accurate knowledge of the whole domain of medicine is too vast to be acquired by one mind, specialties have become a necessity to human progress.

As it is impossible to draw a line of demarcation between the different specialties, or circumscribe the duties of any of them, it frequently becomes a question of great importance with the specialist when to seek the aid of some co-laborer in another department; and this must be particularly so with the general practitioner accustomed as he is to receive all cases that present themselves for treatment; and yet the unassuming and honest general practitioner of medicine would not presume that he was as well acquainted with the numerous conditions of the eye as the oculist, or of the ear as the aurist, still many have an abiding faith that they can extract teeth with the old turnkey as skillfully as the dentist with his forceps specially devised for each individual tooth.

The physician is very promptly and properly consulted in all cases of structural or functional derangement of the mouth, face and adjacent parts; but it does not follow that he should treat all cases that are presented to him; on the contrary, he discharges the highest duty incumbent on him when he takes a specialist into consultation, or directs the patient to some member of the profession who he knows has made such cases a special study.

In former times the general practitioner was constantly required to treat diseases of the teeth, and in some rural districts at the present time his services are still needed. His practice is confined to extracting, or the application of some anodyne remedy to the carious tooth, which permits the caries in a short time to become necrosis, the necrosis in most cases extending to the alveolus, producing abscess and finally resulting in the loss of one or several teeth.

How different the result would have been in the hands of a dentist! Had the case been placed in his care the tooth would have been properly treated and filled with the proper filling, and preserved for an indefinite time.

In all cases where the antrum is affected the dentist should be consulted, for the cause of abscess in the region may be due to an abscessed root or to the malposition of a sound tooth.

Obstinate ulceration of the tongue may be due to a slightly crenated tooth, and this crenated condition of the tooth may be due to decay or a minute mechanical clevage of the enamel. After treating such a case for a long time, the physician discovers the cause of the disorder, and at once removes the offending tooth and of course the ulceration subsides, and he feels unduly elated! But the more careful physician would have sent this patient to a dentist who would either have removed the tooth or repaired it, and thereby cured the ulcerated tongue.

It is no uncommon thing for the general practitioner to treat facial neuralgia for a long time without giving adequate relief. The cause of this disease of the nerves of the head, especially the divisions of the fifth pair, are various and can most always be traced to some local irritation; a crowded or carious condition of the teeth is one of the most fruitful causes.

A short article copied from one of the dental journals will illustrate this statement: "A young man of some twenty-four years of age had been treated by a physician at intervals of several months for facial neuralgia. He was suffering great pain at the time on the right side of his face, his general health was very good; in fact he was a stout, robust, hearty man; the physician stated that he had examined the teeth but could find no defective ones, and referred him to a dentist for a more critical examination. The teeth were large, well developed, clean and even. There were, however, *three* molars and one bicuspid on each side of the lower jaw, the molars and bicuspids in the upper jaw being normal. After careful examination it was decided that the first two molars on the lower jaw were deciduous, although to all appearances, as to size and sharpness of outline they were permanent. The patient stated that while he felt no pain in the teeth, a few days before the neuralgic pains had commenced, he felt that it produced an egreeable sensation to close his teeth tight together, further than this he experienced no local trouble. The two first molars were extracted; they were the second deciduous molars developed to an extraordinary degree. I saw the patient about a year afterwards and there had been no return of the pain, the wisdom teeth had made their appearance and the space made by the removal of the two temporary teeth had nearly closed, leaving no room for the undeveloped bicuspids which remained in the alveolus arrested in their growth by the solidity and long retention of the deciduous teeth."

There is no portion of the body so subject to, or so liable to varied diseases as the maxillary bones, and there is no part of the system that exercises so great an influence over the rest of the body, either in health or disease, as the oral cavity. This will appear more apparent when we reflect on the many protecting canals these bones give to a vascular and nervous apparatus, more complicated in structure, and sustaining a greater degree of organic activity than most other portions of the living skeleton. The presence in the jaws of those dental organisms which undergo successive revolutions, beginning in early foetal life, and ceasing only with the death of the individual, is a constant cause of disturbance, the duration or intensity of which is in proportion to the difficulties and irregularities of their development. There are also morbid conditions of the teeth that are very obscure and difficult of diagnosis, but are none the less potent to produce persistent and extensive nervous disturbances. As may be seen in exostosis of the roots and the formation of calcific nodules in the pulp cavity of the teeth, and to these may be added all those morbid changes to which the tooth substance is liable during the various phases of its growth. The mucous membrane lining the mouth is of considerable thickness, containing numerous glands, and is covered with many tiers of tesselated cells, into the deep surface of which sensitive and vascular papillae project from the membrane itself. It is divided by the alveoli and teeth into an inner and outward space, and forms an elastic and dilatable sack ; within this the rows of teeth can be separated from each other with the lips closed, and much further when the mouth is opened ; it is reflected on the outer surface of the bone and ends on the edges of the alveoli as gum. It is so elastic that when the mouth is open to its widest extent, it is capable of further extension, whilst, when the mouth is closed, it presents no folds. It is clear that as soon as the elasticity of this dilatable sack on either side is impaired, the mobility of the jaw ceases. It is the vestibule of the alimentary canal, and within the radius of a few inches of this cavity all the special nerves are clustered. Now where in the human body is the sense of touch so exquisitely developed as in the ruddy lips and flexible tongue ; the palate and the tongue monopolize the sense of taste, and the sense of smell stands as an advance guard to reject all improper food before the lips, tongue or palate come in contact with it. Three special sentinels are constantly employed in discriminating between suitable and unsuitable substances. Protected as the mouth is from the intrusion of injurious substances, it nevertheless contains within its own walls all the elements necessary for the destruction of its own tissues ; the secretions of the mouth, even when in a healthy condition, uniting with the food in the process of mastication, forms compounds, the residum of which, if allowed to remain in the many interstices present in the oral cavity, threaten the continuity of the teeth. This disintegration of the tooth substance, when once commenced and allowed to go unchecked, increases at each successive step, compounding and re-compounding destructive elements until the soft tissues of the mouth become involved. In all civilized communities sanitary laws with sanitary boards to enforce them are deemed indispensible for the general good; for it is a well established fact that *emanations* from decomposed matter produce pestilence by polluting the atmosphere. If this is true of districts and *communities*, how shall we estimate the injury done to the system of an individual who at every inspiration that comes to his lungs the poisonous *emanations* from the decomposing processes constantly present in a neglected or uncleanly mouth? In the practice of climatic therapeutics, how irrational that physician would be considered who would send his patient to a mountainous district for pure, fresh air, with a mouth containing as much disease and death as the famous box of Pandora. It is very clear that in all low forms of fever the mouth should be kept scrupulously clean by the constant use of the proper materials. It is of the utmost importance in the treatment of syphilis and syphiloid diseases that the hygiene of the mouth receive special attention, for lesions of the throat and mouth present some of the most obstinate forms of these diseases. All crenated teeth should be made smooth, roots of teeth extracted, salivary calculus removed and decayed teeth filled. If the patient is to undergo a long course of mercurial treatment, the teeth should be filled with a non-metalic substance, as any of the metals used in filling teeth would amalgamate more or less with the mercury taken into the system. After recovery the temporary non-metallic filling should be removed and permanent gold fillings substituted. If these precautions are observed, the patient will escape much painful annoyance during the presence of mucous patches, the disease will be easier controlled and will yield more readily to the influence of remedies.

There are few tumors or other affections of the gums that come under the observation of the general surgeon or physician, except those extreme cases that have been growing for years, or have assumed a malignant form. Warty and muscular tumors, polypus, epulis and hypertrophy of the gums are frequently seen by the dentist long before the patient is aware of their existence, and can be treated or removed by him with little inconvenience.

These tumors are not often present even in an incipient stage, in a clean mouth. Where early extirpated and the mouth restored to a cleanly condition, they show no tendency to return. The history of these tumors agrees with their histology—recurring locally as long as the circumstances under which they arose are maintained. They are destroyed by the removal of their local *nidus*, without infecting the system.

In all cases of fracture of the lower jaw, either simple, compound or comminuted, and especially in those cases where the teeth are lost or the occlusion is faulty the case becomes further complicated, and an interdental splint is absolutely necessary to secure a good result.

From these considerations it will be seen that the dentist is as intimately connected with the science of medicine as many other specialists and his services can be called into requisition oftener, and be made a more valuable auxiliary to the general practitioner than most other specialists.

Much has been written and said in regard to the mechanical character of dentistry; and a great deal of this is true; for to be a good dentist, one must not only understand the laws of mechanics, but must use them. His mechanical ingenuity is likely to be tested to its utmost tension in daily practice. If there is any latent genius for the fine arts in the dentist's composition, I know of no better place to improve or test it than in constructing and adapting a full set of teeth for the human mouth.

The construction of artificial teeth requires more skill than the mere mechanic possesses. Such operations to be performed successfully and safely must be preceded by a thorough knowledge of the parts and of the material employed, and this knowledge can only be acquired by a professional education. An acquaintance with the principles and practice of medicine is necessary to prepare the mouth, and to determine when it is in a proper condition to receive artificial work. In the adaptation of artificial teeth we have to deal with living structures, and ignorance on the part of the specialist of the region of the body to which his treatment is confined is to the highest degree *culpable* and should under no circumstances be tolerated. There are other qualifications requisite in the construction of substitutes to supply the loss of the natural teeth, which tax the artistic skill of the dentist to no inconsiderable extent. I refer to that judgment necessary to a proper selection and arrangement of the teeth so as to preserve or restore the expression of the features. The form and character of the teeth in relation to the temperaments, as well as the individual tooth, must be considered; the length, color and size, and the length of the teeth properly divided between the upper and lower set, with a consistent irregularity in the arch.

The demand for cheap artificial teeth has been supplemented by an equal demand for cheap filling material, and it is no uncommon occurrence for persons who enjoy the expensive luxury of wearing diamond jewelry to request the dentist to use cheap material in treating their teeth. The milliner, tailor, dressmaker and jeweler are patronized to the fullest extent, for with the majority of mankind external adornment is of the first and most vital importance. Most people who visit a dentist's office for the first time do so for the same reason that they call a physician when they are in trouble, for they never think of defective teeth, or a diseased condition of the mouth, until they suffer pain or inconvenience. To visit a dental office for the first time without being driven there by dire necessity, or consult a dentist in regard to the condition of children's teeth before the household has been disturbed by their cries, require a great advance in civilization, or at least a higher appreciation of the science of dentistry than falls to the lot of even intelligent people. These statements will appear more clearly when we consider the four principle duties of the dental profession.

The first and most important duty of a skillful dentist is to prevent decay. The second is to stop or arrest decay. The third, to remove offensive teeth from the jaws, and bring the mouth into a healthy condition, and the fourth and last care, to replace the lost teeth by artificial means.

DISCUSSION.

Dr. N. S. Hoff: The essayist takes the ground that dentistry is one of the specialties of general medicine. We do not wish to discuss this question here, but there is a wide-spread opinion that dentistry is not a medical specialty in every sense. There is no question but that medical science lies at the foundation of dental practice, but dentistry as a practice is widely different from medicine as a practice, each one has its peculiar and characteristic functions and these are often quite distinct as well as far from each other. There is no question but that the general medical practitioner can be of the highest value to the dental practitioner, and the relations of the two should be one of intimacy. Frequent consultations with the patient's family physician are of the greatest service. The dentist may learn much from the physician, and the physician may get some benefit in looking on from the dentist's outlook or view point.

Dr. T. G. Rix: Physicians frequently overlook very serious conditions because of lack of knowledge of the results of diseased teeth. Several months ago my attention was called to the case of a young man who had undergone a successful operation for empyemia of the frontal sinuses, but the operation had left the boy blind in one eye. On examining the case I found that the upper first molar on the affected side had been treated and filled, but there was a suspicious appearance about the tooth. I had an X-ray picture made of that side of the face which revealed the presence of an opaque condition in the antrum. The tooth was extracted and an opening made into the antrum, which was found filled with a serous and bloody fluid. This was washed out with antiseptic washss and the antral condition treated. In three weeks time the eyesight had been restored and the antral difficulty was well under way to a cure. I find few remedies so effective for the preliminary treatment of antral troubles as aromatic sulphuric acid.

Dr. S. W. Honey related a case of antral disease which had passed through treatment by three other dentists and physicians, covering a period of two years. He had treated it for four months with varying success. The patient was now taking systemic treatment for catarrh and this seems to be helping on the treatment used locally for the antral trouble.

Dr. P. A. Armstrong had treated a case of suppurating antrum successfully with an irrigating wash of warm dilute salt water, followed by a peroxid of hydrogen injection, or a dilute solution of chlorid of zinc.

Dr. J. I. Backus: I find great difficulty in getting patients of this kind to give constant and proper attention to the use of remedies as prescribed.

Dr. A. L. LeGros: I have a system of keeping in touch with such, and in fact with all my patients. I use the system of card indexes, and call these patients into my office when I want to see them. I find that it pays to keep in contact with patients in this way.

